# Coronary artery calcium scoring for cardiovascular risk stratification: A prospective cohort study

**DOI:** 10.6026/973206300222010

**Published:** 2026-04-30

**Authors:** Elangovan Raman, Aishwarya Kankipati Sailaxmi, Amoolya Rao Amaravadhi, Sophia Aldrine Suresh, Manvitha Bachala, Jyoti Saikia, Kosmita Karki

**Affiliations:** 1 Department of Internal Medicine, Institute Of Internal Medicine, Madras Medical College, Chennai, Tamil Nadu, India; 2 Depertment of Medicine, Ramaiah Medical College, Bengaluru, Karnataka, India; 3 Department of General Medicine, Malla Reddy Institute of Medical Sciences, Hyderabad, Telangana, India; 4 Department of Medicine, Evya Multispeciality Hospitals, Hyderabad, Telangana, India; 5 Department of Medicine, Amara Hospitals, Tirupati, Andhra Pradesh, India; 6 Department of Internal Medicine, Apollo Hospitals, International Hospital, Guwahati, Assam, India; 7 Department of Medicine, Manipal College of Medical Sciences, Fulbari, Pokhara - 11 , Kaski 33700, Nepal

**Keywords:** Coronary artery calcium (CAC), cardiovascular risk, atherosclerosis, computed tomography, coronary artery disease (CAD), risk stratification, major adverse cardiovascular events

## Abstract

Traditional cardiovascular risk models fail to detect subclinical atherosclerosis in a substantial proportion of asymptomatic individuals. Therefore, it is of interest to evaluate the prognostic value of coronary artery calcium (CAC) scoring in predicting major adverse cardiovascular events over five years. Higher CAC categories were associated with progressively increased myocardial infarction, stroke and cardiovascular mortality, with CAC ≥400 demonstrating the highest event burden. Each 100-unit increase in CAC score independently increased cardiovascular risk nearly twofold (HR 1.92, p=0.001). Thus, addition of CAC scoring to traditional risk models significantly improved risk stratification with a net reclassification improvement of 0.24.

## Background:

Cardiovascular disease remains the leading cause of mortality worldwide despite advances in pharmacologic and interventional therapies [1]. Early identification of individuals at high risk is essential to guide preventive strategies and reduce long-term morbidity [2]. Traditional cardiovascular risk assessment models such as the Framingham Risk Score and ASCVD calculator rely on demographic variables and conventional risk factors, including hypertension, diabetes, dyslipidemia and smoking [3]. However, these models estimate probability rather than directly measuring atherosclerotic burden and may underestimate risk in certain individuals [4]. Atherosclerosis develops silently over decades before clinical manifestation. Subclinical plaque accumulation may be present even in individuals categorized as low or intermediate risk by traditional models [5]. Coronary artery calcium scoring obtained through non-contrast computed tomography provides a direct and quantitative assessment of calcified coronary plaque [6]. The Agatston score reflects cumulative plaque burden and has demonstrated strong correlation with total coronary atherosclerotic load [7].

Multiple studies have shown that increasing CAC scores are associated with progressively higher risks of myocardial infarction, stroke and cardiovascular mortality independent of traditional risk factors [8]. A CAC score of zero is associated with very low short-term cardiovascular event rates, whereas higher categories, particularly ≥400 Agatston units, indicate substantial atherosclerotic burden and elevated event risk. Importantly, CAC scoring has been proposed as a tool for reclassifying individuals in intermediate-risk categories and guiding initiation of statins or other preventive therapies [9]. Therefore, it is of interest to evaluate the association between CAC score categories and incident major adverse cardiovascular events over five years and to determine the incremental prognostic value of CAC scoring when added to traditional cardiovascular risk models in a prospective cohort.

## Materials and Methods:

This prospective cohort study enrolled asymptomatic adults undergoing coronary artery calcium (CAC) scoring as part of routine cardiovascular risk evaluation in preventive cardiology clinics. Individuals with known coronary artery disease, prior myocardial infarction, prior revascularization, heart failure, or chronic inflammatory disorders were excluded to avoid confounding due to established atherosclerotic disease. Baseline assessment included demographic characteristics and traditional cardiovascular risk factors such as hypertension, diabetes mellitus, dyslipidemia, smoking status and family history of coronary artery disease. Biochemical parameters including fasting glucose, lipid profile and high-sensitivity C-reactive protein were recorded. Coronary artery calcium scoring was performed using non-contrast multidetector computed tomography. CAC scores were quantified using the Agatston method and categorized as 0, 1-99, 100-399 and ≥400 Agatston units. These categories reflected absent, mild, moderate and severe calcification, respectively. Participants were followed for a minimum of five years. The primary outcome was occurrence of major adverse cardiovascular events (MACE), d efined as myocardial infarction, stroke, cardiovascular death, or coronary revascularization. Events were confirmed through hospital records, physician documentation and death certification where applicable. Event-free survival was analyzed using Kaplan-Meier survival curves. Independent predictors of MACE were identified using multivariable Cox proportional hazards regression analysis. The incremental predictive value of CAC scoring beyond traditional risk factors was evaluated using net reclassification improvement (NRI). A p-value <0.05 was considered statistically significant. Ethical approval was obtained from the institutional review board and informed consent was secured from all participants prior to enrollment.

## Results:

A total of 130 asymptomatic adults were included in the final analysis and completed five-year follow-up. Participants were distributed across CAC categories as follows: CAC = 0 (n=40), CAC 1-99 (n=35), CAC 100-399 (n=30) and CAC ≥400 (n=25). Increasing CAC category was significantly associated with older age and higher prevalence of traditional cardiovascular risk factors. Mean age increased progressively from 52.3 years in the CAC = 0 group to 65.4 years in the CAC ≥400 group (p=0.002). The prevalence of hypertension, diabetes, dyslipidemia, smoking and family history of coronary artery disease rose significantly across increasing CAC categories (all p<0.05). Severe calcification (CAC ≥400) was associated with the highest burden of metabolic and lifestyle risk factors. Major adverse cardiovascular events demonstrated a graded increase with rising CAC scores. MACE incidence was 2.5% in the CAC = 0 group and increased to 35.0% in the CAC ≥400 group. Myocardial infarction, stroke and cardiovascular mortality followed a similar progressive trend. Individuals with CAC ≥400 experienced the highest event rates across all outcome categories. Kaplan-Meier survival analysis showed a marked decline in five-year event-free survival with increasing CAC burden. Event-free survival was 98% in the CAC = 0 group compared with 63% in the CAC ≥400 group (p=0.001). Survival curves separated early and remained significantly divergent throughout follow-up. Multivariable Cox regression analysis identified CAC score as an independent predictor of MACE after adjustment for age, hypertension, diabetes, dyslipidemia and smoking. Each 100-unit increase in CAC score was associated with a nearly twofold increase in cardiovascular event risk (HR 1.92, 95% CI 1.55-2.30; p=0.001). Traditional risk factors remained significant predictors, but CAC demonstrated the strongest magnitude of association. Addition of CAC scoring to traditional risk models significantly improved risk classification. Net reclassification improvement was 0.24 (p=0.001) and 22% of individuals were correctly reclassified into more appropriate risk categories. These findings demonstrate that CAC scoring enhances prognostic precision beyond conventional risk assessment alone. Table 1 (see PDF) shows a progressive increase in age and prevalence of hypertension, diabetes, and dyslipidemia, smoking and family history of coronary artery disease with increasing CAC score categories, with mean CAC rising from 0 to 520.6 across groups. Table 2 (see PDF) demonstrates a graded rise in MACE incidence from 2.5% in CAC = 0 to 35.0% in CAC ≥400, with corresponding increases in myocardial infarction, stroke and cardiovascular mortality rates. Table 3 (see PDF) indicates that CAC score is the strongest independent predictor of MACE, with each 100-unit increase associated with a hazard ratio of 1.92, exceeding the magnitude of traditional risk factors. Table 4 (see PDF) highlights that addition of CAC scoring improved risk stratification, correctly reclassifying 22% of individuals with a net reclassification improvement of 0.24. Table 5 (see PDF) shows a marked decline in five-year event-free survival from 98% in CAC = 0 to 63% in CAC ≥400, demonstrating a strong inverse relationship between calcification burden and survival.

## Discussion:

This prospective cohort study demonstrates a strong graded association between coronary artery calcium burden and five-year incidence of major adverse cardiovascular events in asymptomatic adults [10]. Cardiovascular event rates increased progressively across CAC categories, with severe calcification identifying individuals at markedly elevated risk. The substantial reduction in event-free survival among those with CAC ≥400 underscores the clinical relevance of quantifying calcified plaque burden [11]. Although traditional risk factors such as age, hypertension, diabetes, dyslipidemia and smoking remained independently associated with outcomes, CAC score showed the highest magnitude of effect [12]. Each 100-unit increase in CAC was associated with a near doubling of cardiovascular risk, indicating that structural plaque burden provides incremental prognostic information beyond clinical risk profiling. This finding highlights the importance of direct atherosclerotic quantification rather than reliance solely on probability-based risk calculators [13]. The pronounced survival advantage observed in individuals with CAC = 0 supports the concept of a "negative risk marker." In contrast, higher CAC categories demonstrated progressively worse survival curves, with early and sustained separation over follow-up. These findings emphasize that calcification burden not only predicts events but also stratifies long-term survival probability [14, 15].

Importantly, the addition of CAC scoring significantly improves risk classification accuracy. The observed net reclassification improvement indicates that a substantial proportion of individuals were reassigned to more appropriate risk categories when CAC data were incorporated. This has direct implications for therapeutic decision-making, particularly in patients categorized as intermediate risk using traditional models [16, 17]. The results reinforce the role of CAC scoring as a structural biomarker of cumulative atherosclerotic exposure. By quantifying coronary plaque burden, CAC scoring refines cardiovascular risk estimation and enables more individualized preventive strategies. Individuals with high CAC burden may benefit from intensified pharmacologic and lifestyle interventions, whereas those with zero CAC may avoid unnecessary escalation of therapy. Limitations include the moderate cohort size and absence of external validation. Larger multicentre studies with extended follow-up are required to confirm long-term predictive stability and evaluate cost-effectiveness of routine CAC integration into clinical practice.

## Conclusion:

Coronary artery calcium scoring is a strong independent predictor of major adverse cardiovascular events and demonstrates a graded association with five-year event risk. Each incremental increase in CAC burden significantly amplifies cardiovascular risk beyond traditional factors and markedly reduces event-free survival. Thus, integration of CAC scoring into cardiovascular risk assessment improves risk stratification and supports more precise preventive decision-making.
